# Supplementary material for: Combi-seq for multiplexed transcriptome-based profiling of drug combinations using deterministic barcoding in single-cell droplets
Source: Nat Commun. 2022 Aug 1;13:4450. doi: 10.1038/s41467-022-32197-0 (PMC9343464; doi:10.1038/s41467-022-32197-0)
Supplement: Supplementary file 1 — Supplementary Information [file 41467_2022_32197_MOESM1_ESM.pdf]

Combi-Seq for multiplexed transcriptome-based profiling of drug combinations using deterministic barcoding in single-cell droplets

Supplementary Information

Mathur L[1,2], Szalai B[3,8], Du NH[4], Utharala R[1], Ballinger M[1], Landry JJM[1], Ryckelynck M[5], Benes V[1], Saez-Rodriguez J[6,7,*], Merten CA[1,4,*]

1. European Molecular Biology Laboratory (EMBL), Meyerhofstr. 1, Heidelberg, Germany
2. Collaboration for joint PhD degree between EMBL and Heidelberg University, Faculty of Biosciences
3. Department of Physiology, Faculty of Medicine, Semmelweis University, Budapest, Hungary
4. Institute of Bioengineering, School of Engineering, École Polytechnique Fédérale de Lausanne (EPFL), Lausanne, Switzerland
5. Université de Strasbourg, CNRS, Architecture et Réactivité de l'ARN, UPR 9002, Strasbourg, France.
6. Faculty of Medicine and Heidelberg University Hospital, Institute of Computational Biomedicine, Heidelberg University, Heidelberg, Germany
7. Faculty of Medicine, Joint Research Centre for Computational Biomedicine (JRC-COMBINE), RWTH Aachen University, Aachen, Germany
8. Present address: Turbine Simulated Cell Technologies Ltd., Budapest, Hungary

These authors contributed equally: Mathur L, Szalai B
* Correspondence: christoph.merten@epfl.ch, pub.saez@uni-heidelberg.de

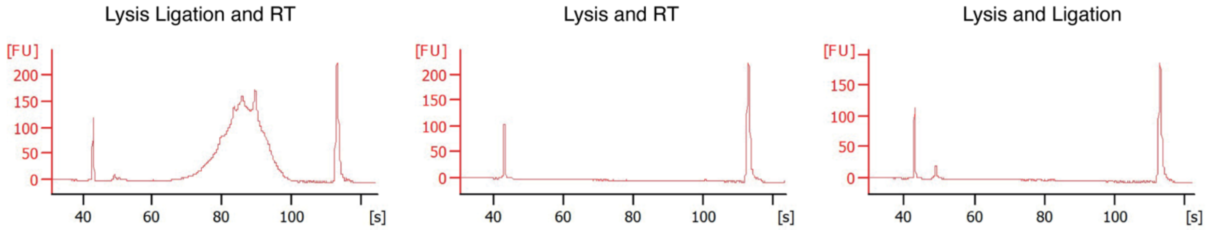

Supplementary Fig. 1: Bioanalyzer traces showing specificity of ligation for the generation of functional barcodes. After picoinjections with the whole reaction mix (cell lysis, ligation and RT, left plot), reagents only for cell lysis and RT (center plot) and reagents for cell lysis and ligation (right plot), cDNA was purified and amplified (see methods) and loaded on a high sensitivity chip in a Bioanalyzer (Agilent). RT: reverse transcription. Source data are provided as a Source Data file.

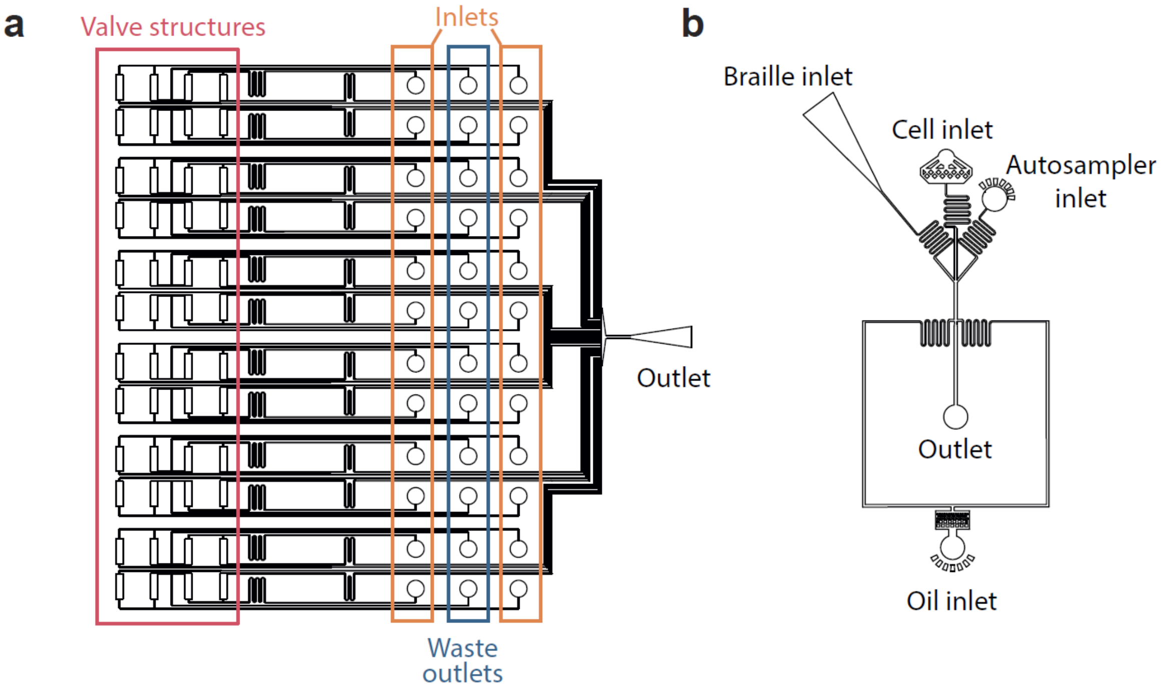

Supplementary Fig. 2: Chip designs. (a) Chip design for the Braille valves. The chips were mounted on top of a Braille display with the rectangular valve structures on top of the pins. Inlets were used to inject drugs which in the default mode were directed to the waste outlets. Drug plugs were generated at the outlet to which a delay tube was connected horizontally into the funnel like structure. **(b)** Droplet maker chip design used to inject drug plugs via the horizontal Braille inlet and drugs from the 96-well plate via the Autosampler inlet. Cells were injected via the central cell inlet and co-encapsulated into droplets at the flow focusing junction by co-injecting oil in the perpendicular channels.

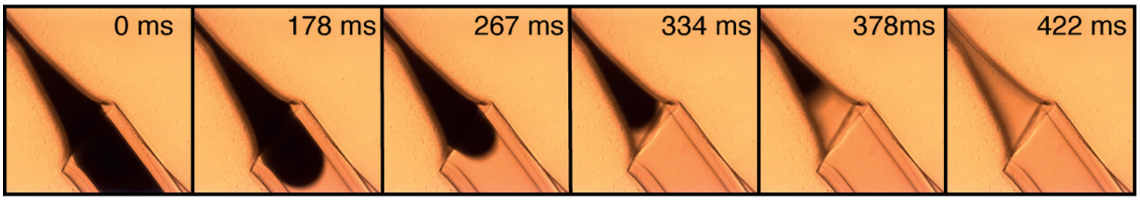

Supplementary Fig. 3: Frames from a video sequence recording the injection of plugs labelled with Trypan Blue. The horizontal inlet port prevents plug breakups getting stuck at the inlet, which are then picked up by subsequent plugs causing cross-contaminations.

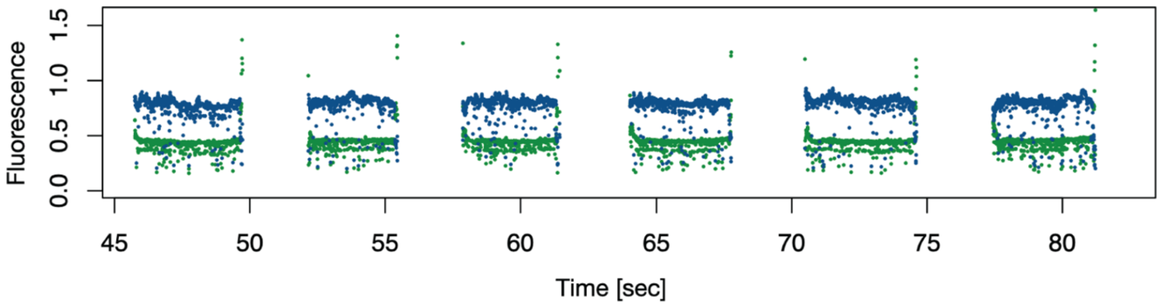

Supplementary Fig. 4: Fluorescence intensities of six combinations generated with green (autosampler) and UV (Braille valves). UV fluorescence increased when plugs were injected and decreased at each end of the plug. Green signals decreased at the beginning of each plug since the continuously injected compounds from the autosampler were diluted. At the end of each plug the intensities increased, due to the end of the plug resulting in a higher concentration of the autosampler compound. Data was filtered for blue positive data points to remove green signals from between injected plugs. Source data are provided as a Source Data file.

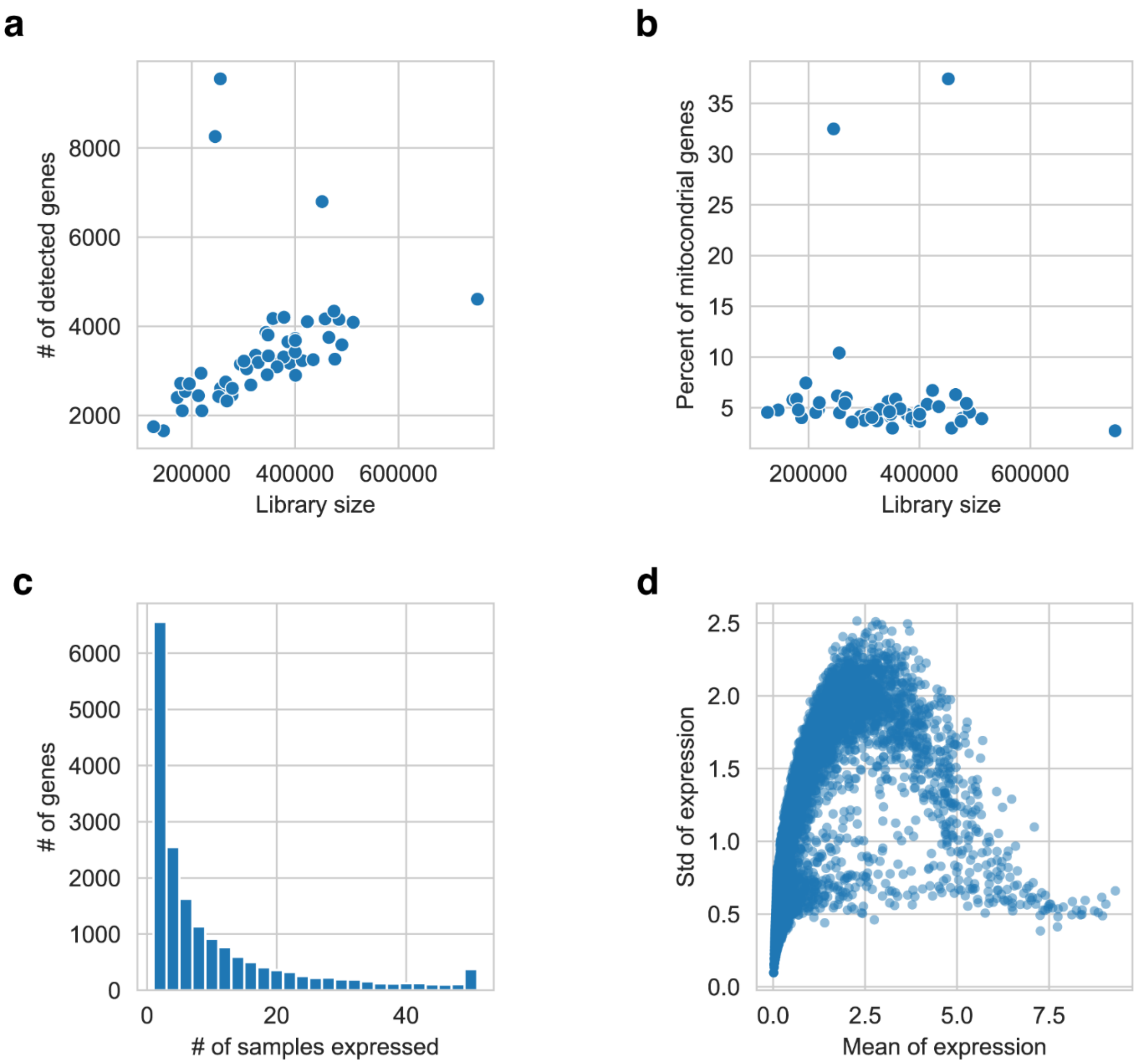

Supplementary Fig. 5: Quality control for small scale drug screen (a) Relationship between the total counts / samples and the number of detected genes / samples. **(b)** Relationship between the total counts / sample and the percent of mitochondrial genes. **(c)** Distribution for the number of samples where a given gene was expressed. **(d)** Mean - Standard deviation relationship for the log1p transformed read counts. Source data are provided as a Source Data file.

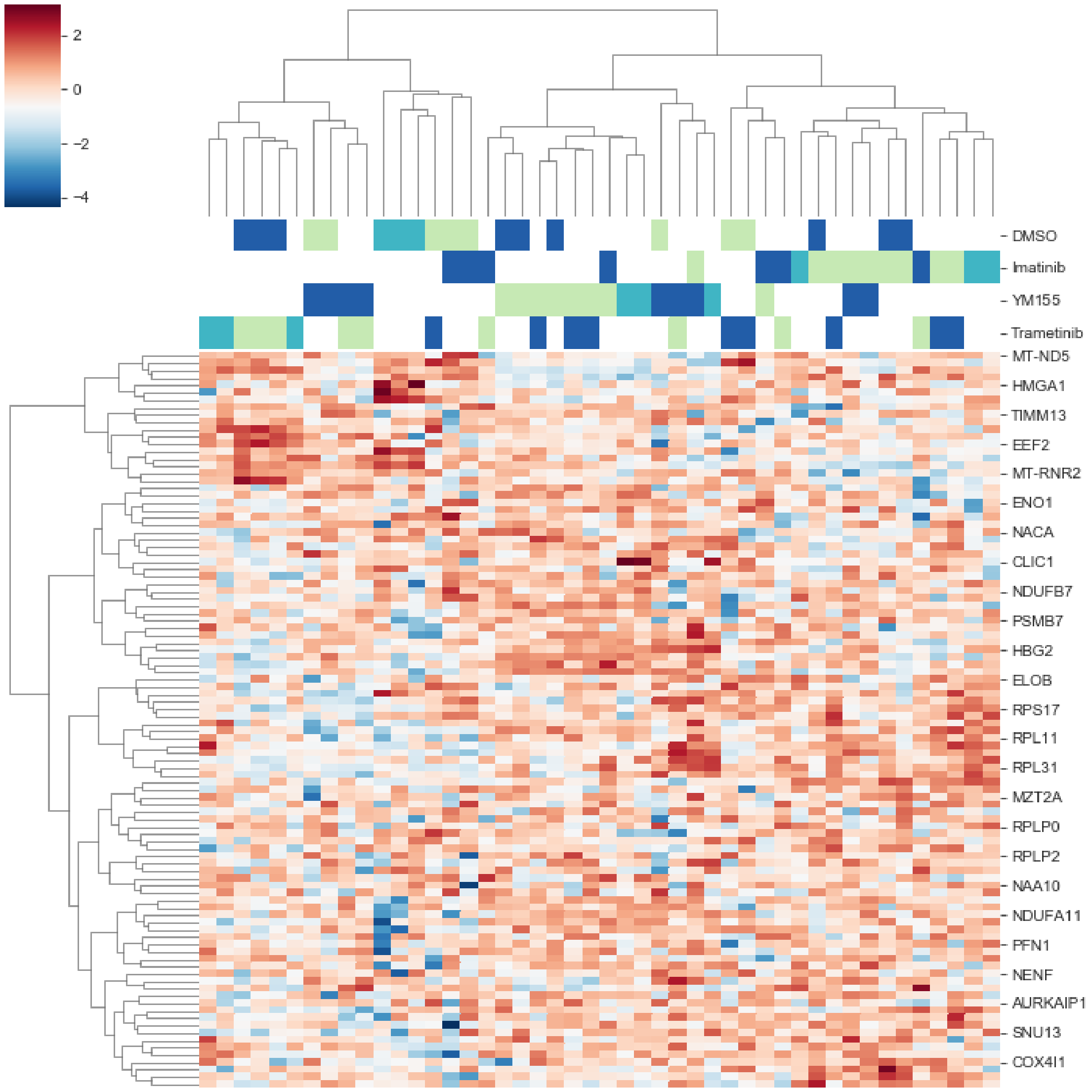

Supplementary Fig. 6: Clustering of the small-screen samples based on the top 100 highly expressed genes. Normalized gene expression values (heatmap color code) were used to perform hierarchical clustering both on genes (x axis) and samples (y axis). Drugs of combinations are color coded (light-green: autosampler drug, blue: braille valves drug, cyan: Same drug from autosampler and Braille valves). Source data are provided as a Source Data file.

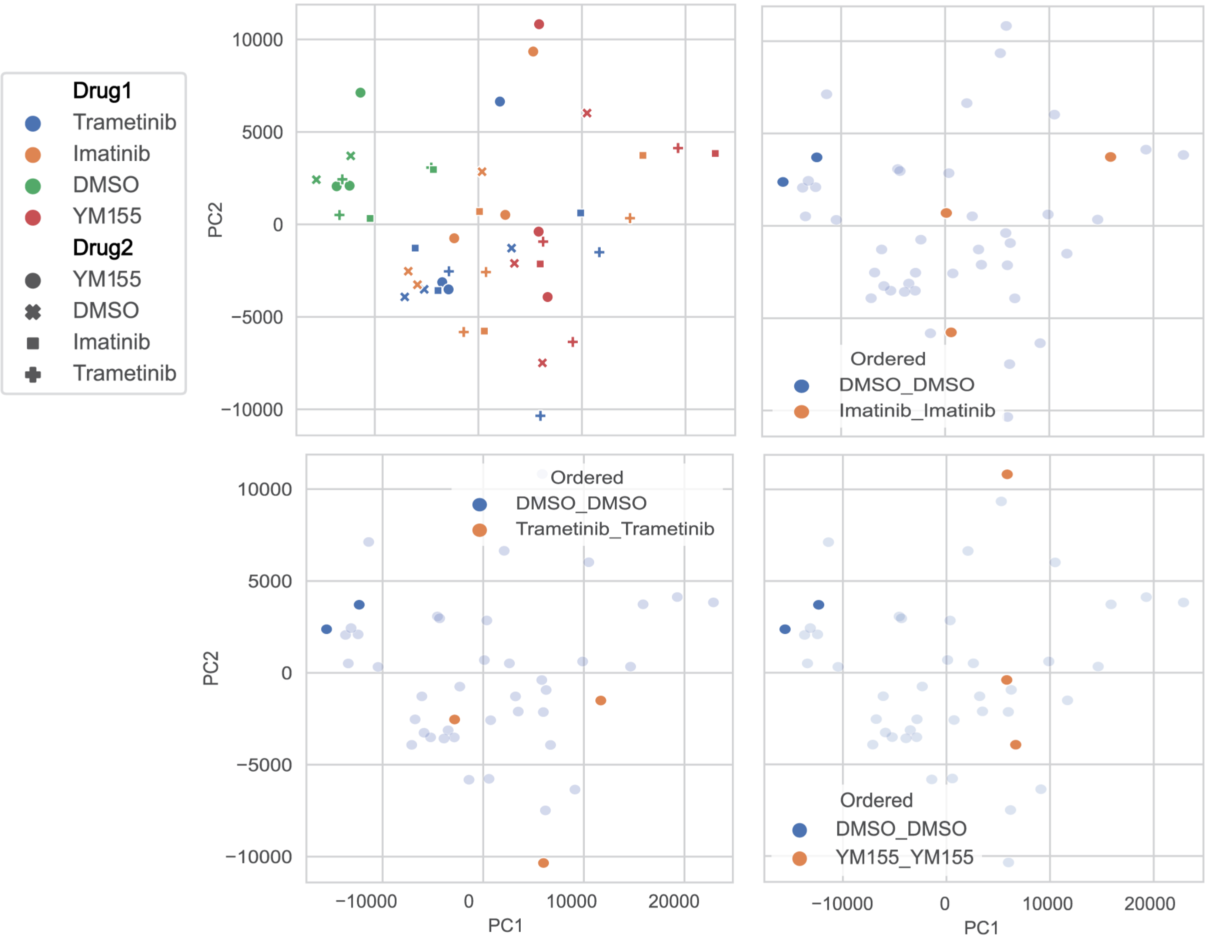

Supplementary Fig. 7: Principal component analysis of the 4x4 screen data, highlighting individual drugs and DMSO controls. Source data are provided as a Source Data file.

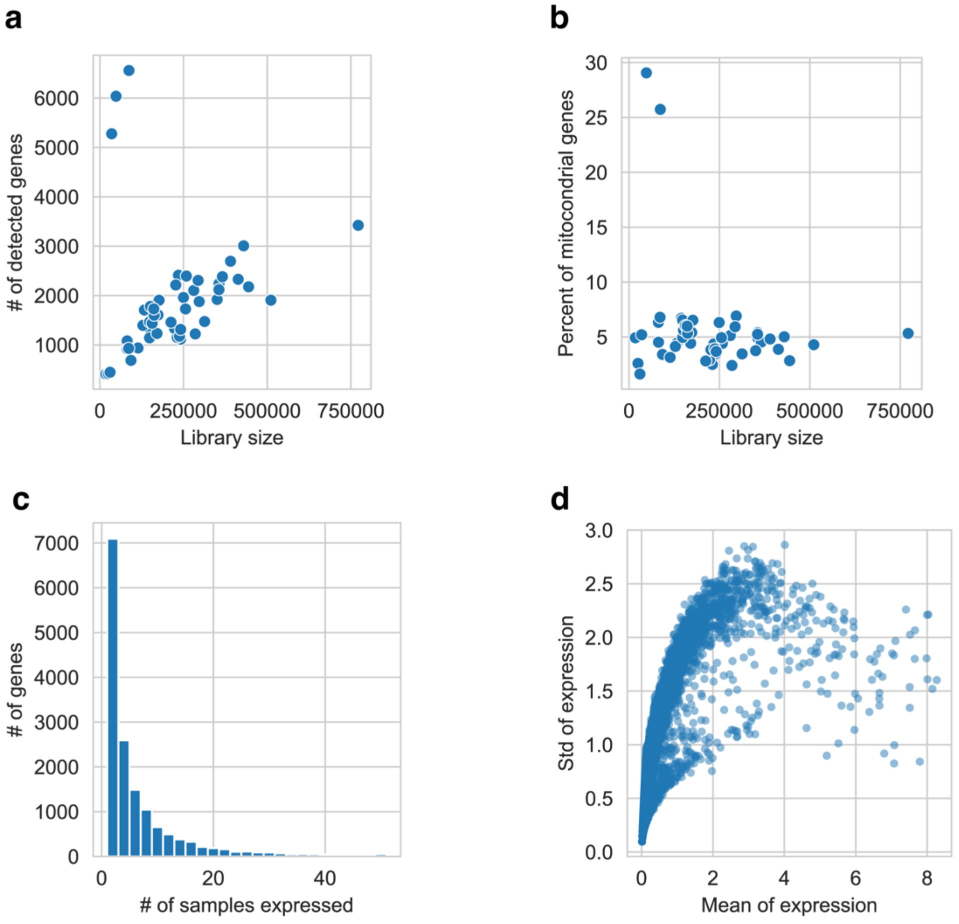

Supplementary Fig. 8: Quality control for small scale drug screen with swapped barcoding mode: (a) Relationship between the total counts / samples and the number of detected genes / samples. **(b)** Relationship between the total counts / sample and the percent of mitochondrial genes. **(c)** Distribution for the number of samples where a given gene was expressed. **(d)** Mean - Standard deviation relationship for the log1p transformed read counts. Source data are provided as a Source Data file.

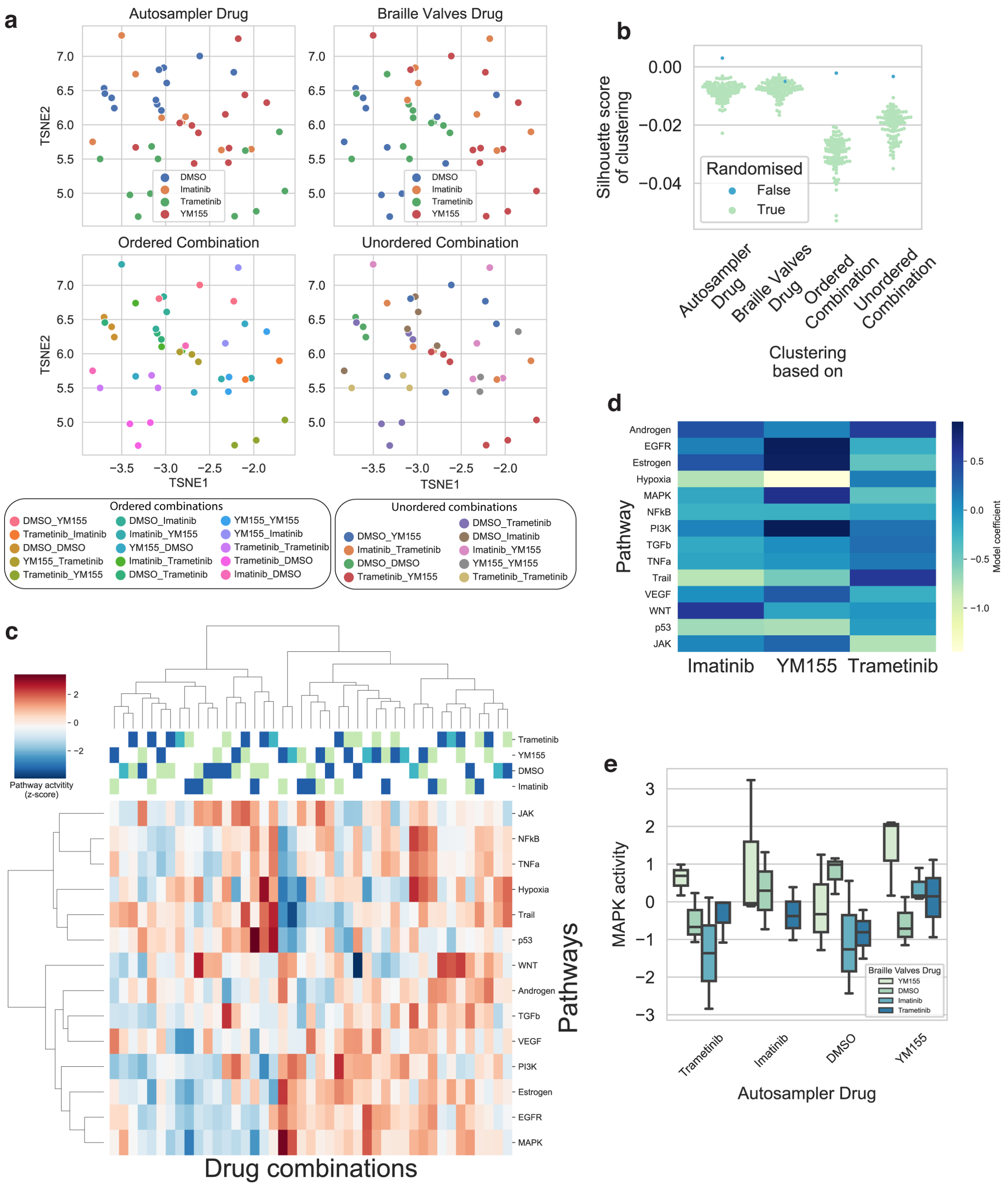

Supplementary Fig. 9: Functional analysis for small scale drug screen with swapped barcoding mode. (A) TSNE plots of normalized gene expression data. Samples are color coded based on Autosampler Drug (top left panel), Braille Valves Drug (top right panel), Ordered Combination (bottom left panel) and Unordered Combination (bottom right panel). Color code is labeled for Autosampler and Braille Valves Drugs (top panels). **(B)** Silhouette scores of sample clustering based on Autosampler / Braille Valves Drugs and Ordered / Unordered Combinations. Silhouettes scores are compared to random distributions (color code) created by permuting sample labels. **(C)** Pathway activity heatmap of samples. PROGENy pathway activities were calculated for each sample (z-scores of pathway activities, color code) and the pathway activity matrix was hierarchically clustered. Drugs of combinations are color coded (light-green: autosampler drug, blue: braille valves drug, cyan:

Same drug from autosampler and Braille valves). **(D)** Drug induced pathway activity changes. Linear model (pathway_activity ~YM155 + Imatinib + Trametinib) was fitted for each pathway, and the linear model coefficients (color code) for each drug is plotted as a heatmap. (**E**) Drug induced MAPK activity changes. MAPK activity (y axis) grouped based on Autosampler Drug (x axis) and Braille Valves Drug (color code), n = 3 biological independent experiments for all samples, except for YM155_Imatinib and DMSO_Imatinib n = 2. The box plots show the median and first and third quartiles as a box, and the whiskers indicate the most extreme data points within 1.5 lengths of the box. Source data are provided as a Source Data file.

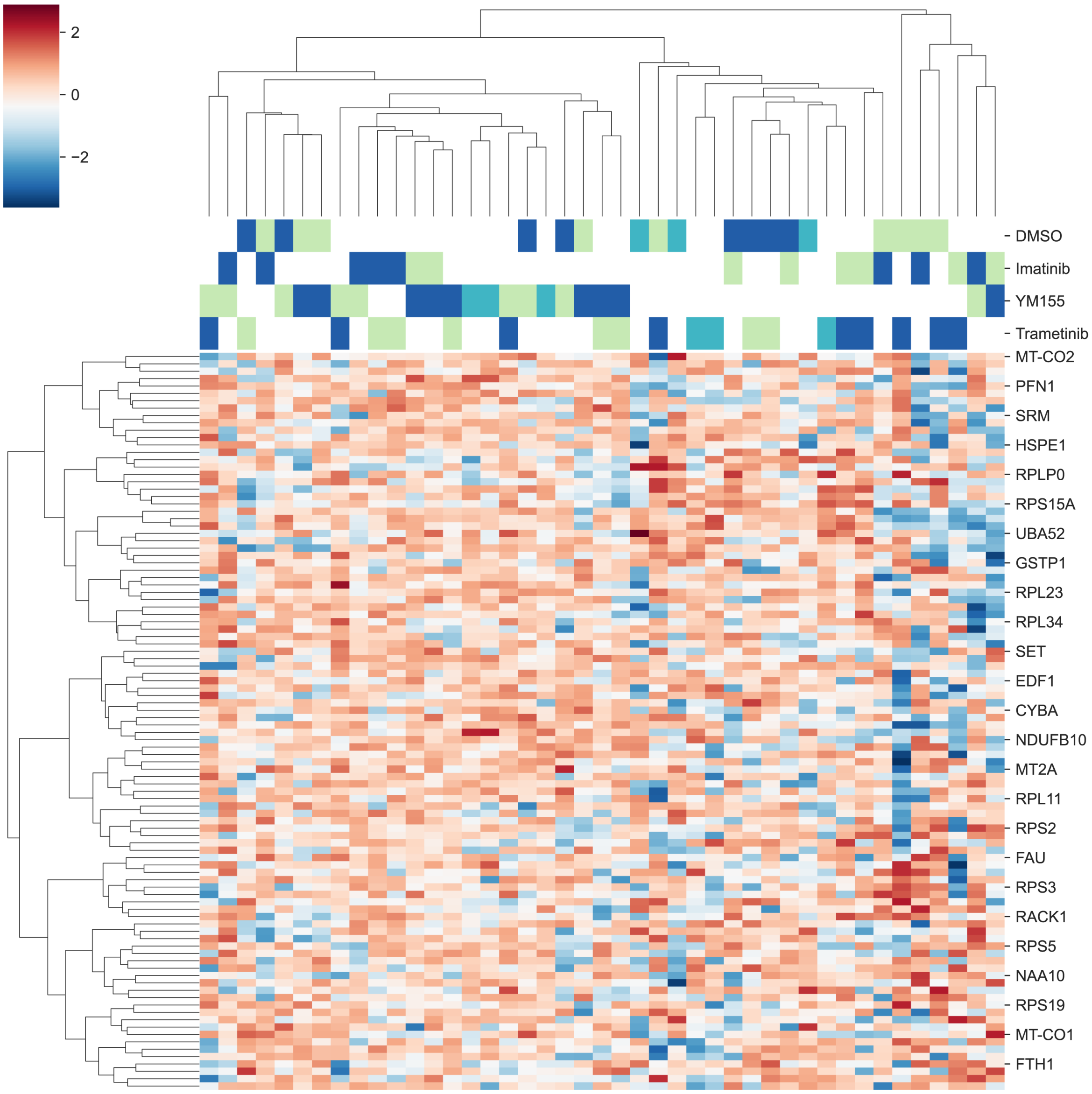

Supplementary Fig. 10: Clustering based on the top 100 highly expressed genes of the small-screen samples with swapped barcodes (Braille valves drugs supplemented with BC-PCR and autosampler drugs supplemented with BC-RT). Normalized gene expression values (heatmap color code) were used to perform hierarchical clustering both on genes (x axis) and samples (y axis). Drugs of combinations are color coded (light green: autosampler drug, blue: braille valves drug, cyan: Same drug from autosampler and Braille valves). Source data are provided as a Source Data file.

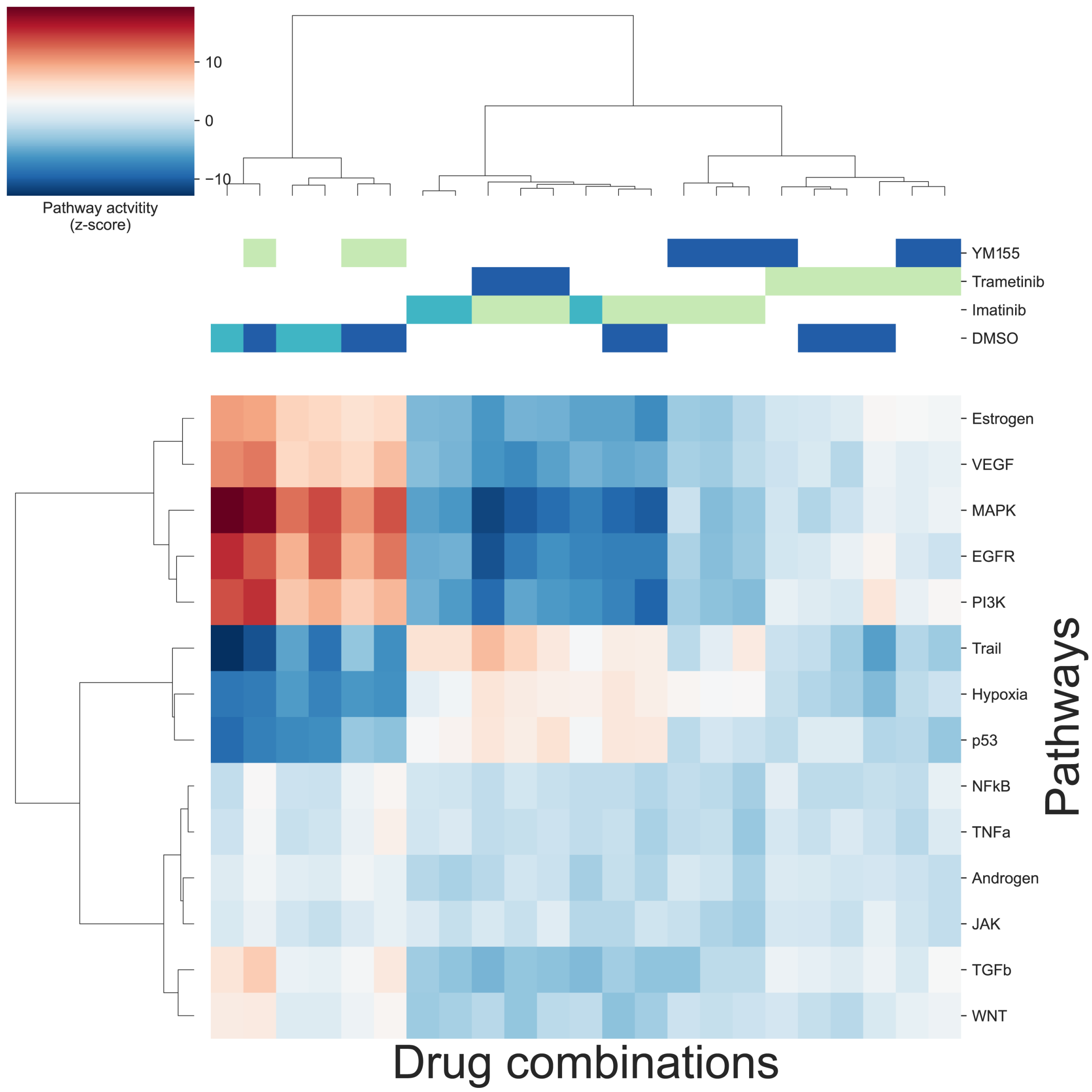

Supplementary Fig. 11: Pathway activity heatmap of bulk high input and deep sequenced Combi-Seq samples: PROGENy pathway activities were calculated for each sample (z-scores of pathway activities, color code blue to red) and the pathway activity matrix was hierarchically clustered. Drugs of combinations are color coded (light green: autosampler drug, blue: braille valves drug, cyan: Same drug from autosampler and Braille valves). Source data are provided as a Source Data file.

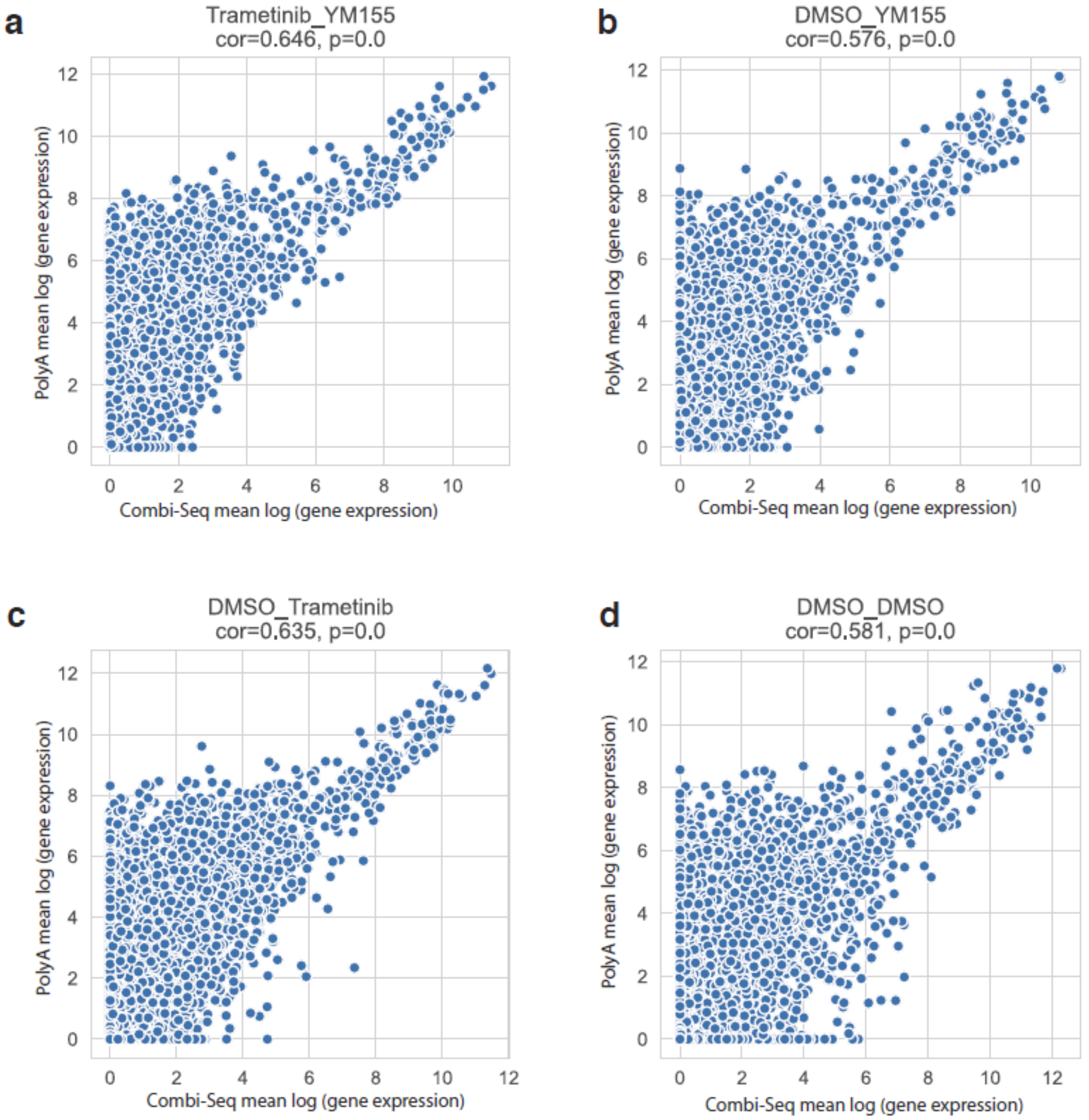

Supplementary Fig. 12: Correlations between bulk Combi-Seq and polyA based normalized gene expression data from (**a**) Trametinib and YM155 (R^2=0.417), (**b**) YM155 and (R^2=0.332), (**c**) Trametinib treated samples as well as (R^2=0.403) (**d**) untreated control samples (R^2=0.338). Pearson correlation was performed without adjustment for multiple comparisons. Source data are provided as a Source Data file.

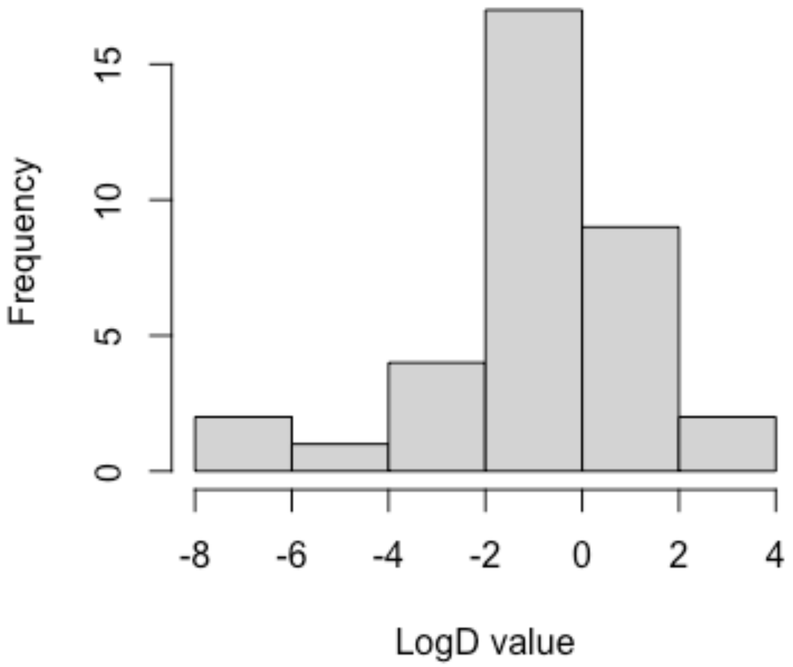

Supplementary Fig. 13: Log D values of the drug panel. Log D values at pH 7.4 of drugs were obtained from the ChEMBL database. Source data are provided as a Source Data file.

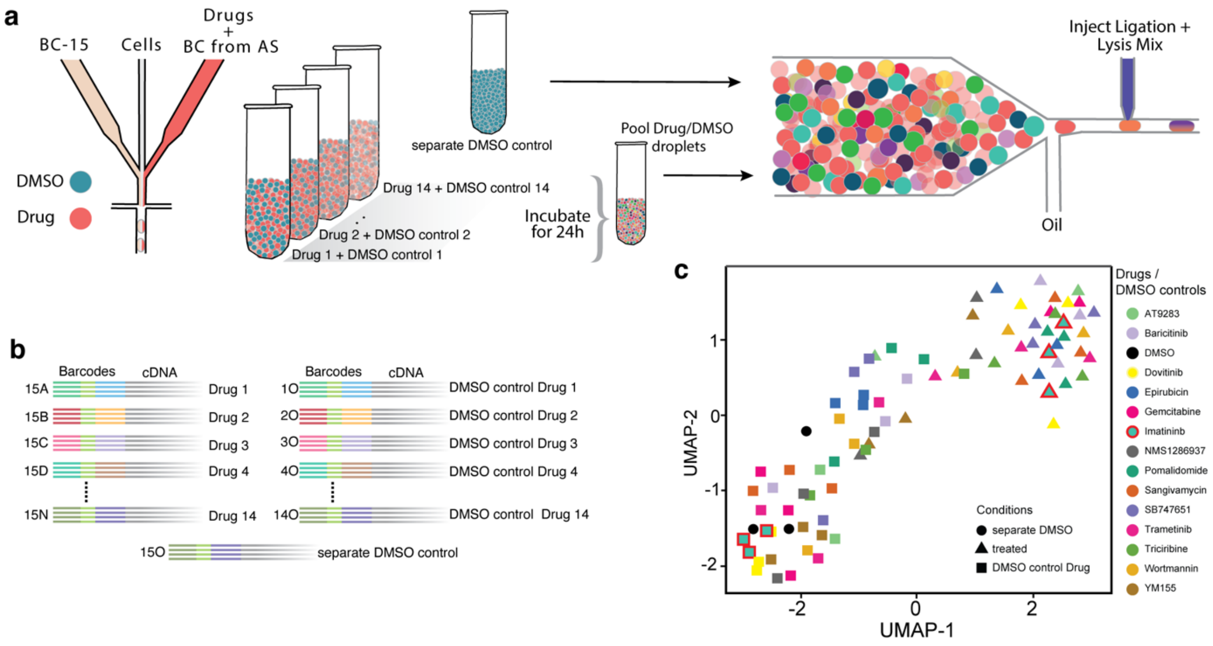

Supplementary Fig. 14: Assessing the effect of potential drug exchange on gene expression. (a) Experimental setup to determine the impact of drug exchange between droplets: Droplets with cells and a single drug (Drug 1-14) or DMSO (DMSO control 1-14) were mixed and incubated for 24h. After the incubation, all emulsion mixtures (Drug 1-14 + DMSO 1-14) were pooled and injected into a device for pico-injection in order to ligate the barcodes and lyse the cells. The same setup was used to generate separate DMSO controls, where only droplets containing DMSO and cells were incubated. These samples were not pooled, but processed separately. **(b)** Samples were used to generate cDNA libraries for sequencing, containing 14 different treatment conditions (Drug 1-14), the corresponding DMSO controls (DMSO control 1-14) that were incubated with droplets containing drugs and additional separate DMSO controls. **(c)** UMAP of all 14 treated samples (triangles), the corresponding DMSO controls (squares) and separate DMSO controls (points), with colors referring to the tested drug. Source data are provided as a Source Data file.

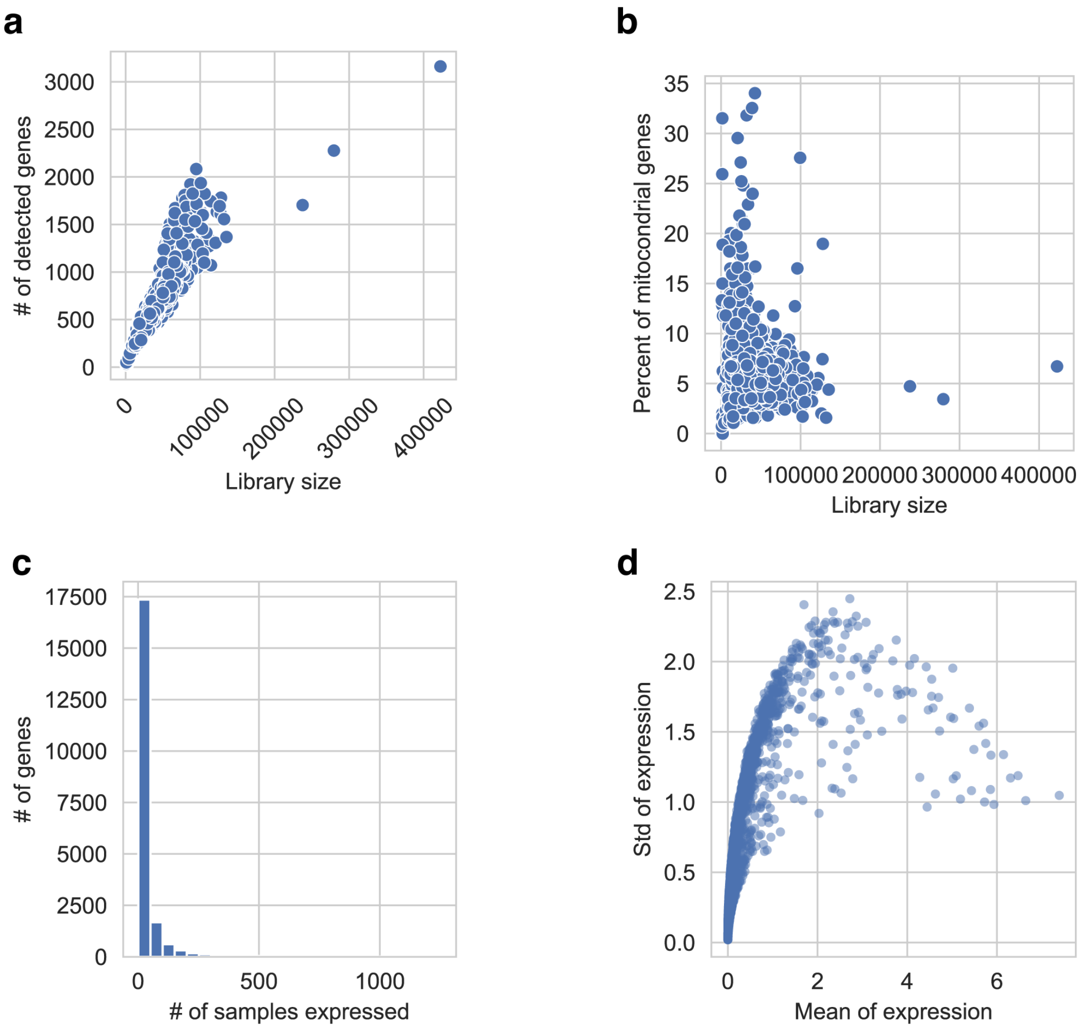

Supplementary Fig. 15: Quality control for large scale drug screen. (a) Relationship between the total counts / samples and the number of detected genes / samples. **(b)** Relationship between the total counts / sample and the percent of mitochondrial genes. **(c)** Distribution for the number of samples where a given gene was expressed. **(d)** Mean - Standard deviation relationship for the log1p transformed read counts. Source data are provided as a Source Data file.

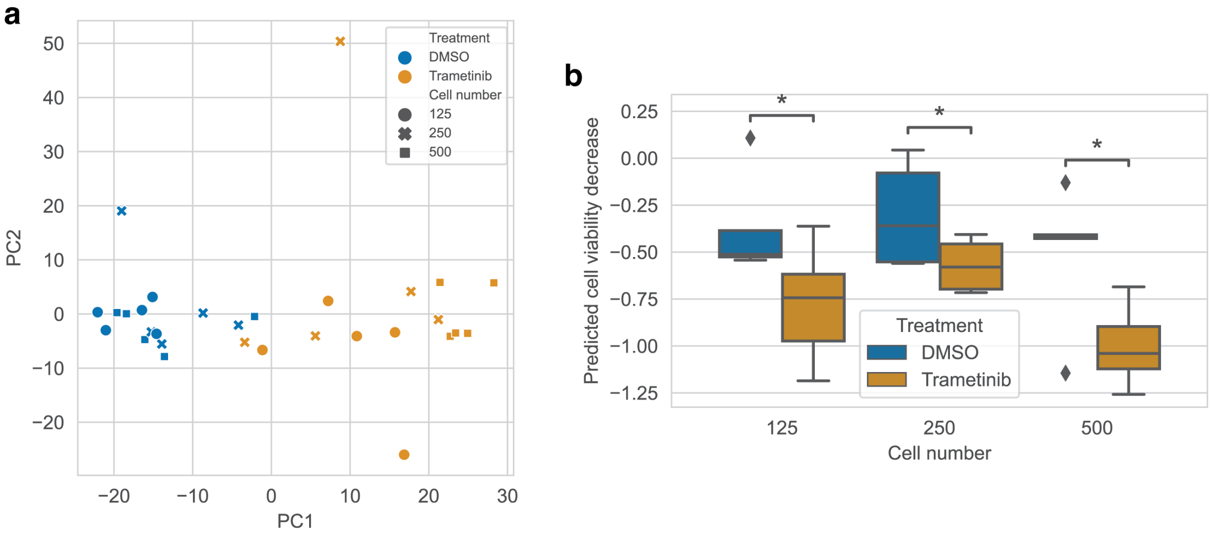

Supplementary Fig. 16: Impact of input cell numbers on variability in gene expression-based prediction of cell viability. (**a**) PCA of Trametinib treated cells and DMSO controls for 125, 250 and 500 input cells. (**b**) Predicted cell viability for trametinib treated and untreated (DMSO) samples for which 125 cells, 250 cells and 500 cells were collected, n = 5 biological independent experiments (* refers to a p-value = 0.048 for all conditions, Mann-Whitney test one-sided). The box plots show the median and first and third quartiles as a box, and the whiskers indicate the most extreme data points within 1.5 lengths of the box. Source data are provided as a Source Data file.

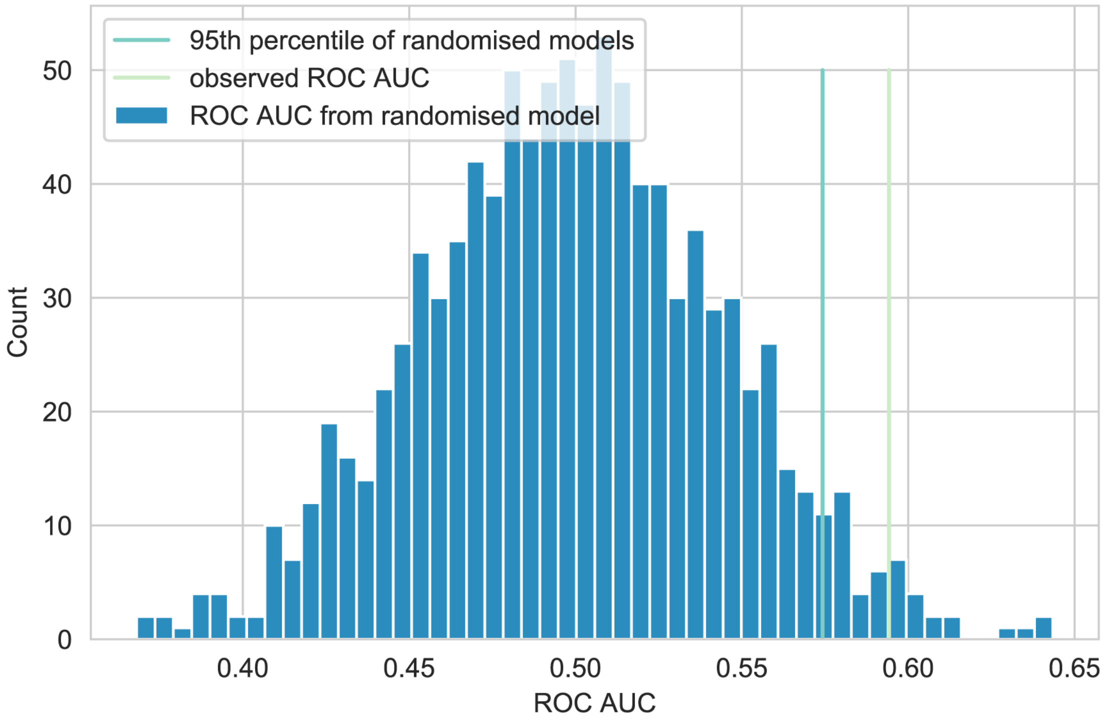

Supplementary Fig. 17: Comparison of a random distribution of ROC (Receiver Operating Characteristics) area under the curves (AUCs), generated by permutating drug labels. The observed AUC was found outside of the 95th percentile of this distribution. Source data are provided as a Source Data file.

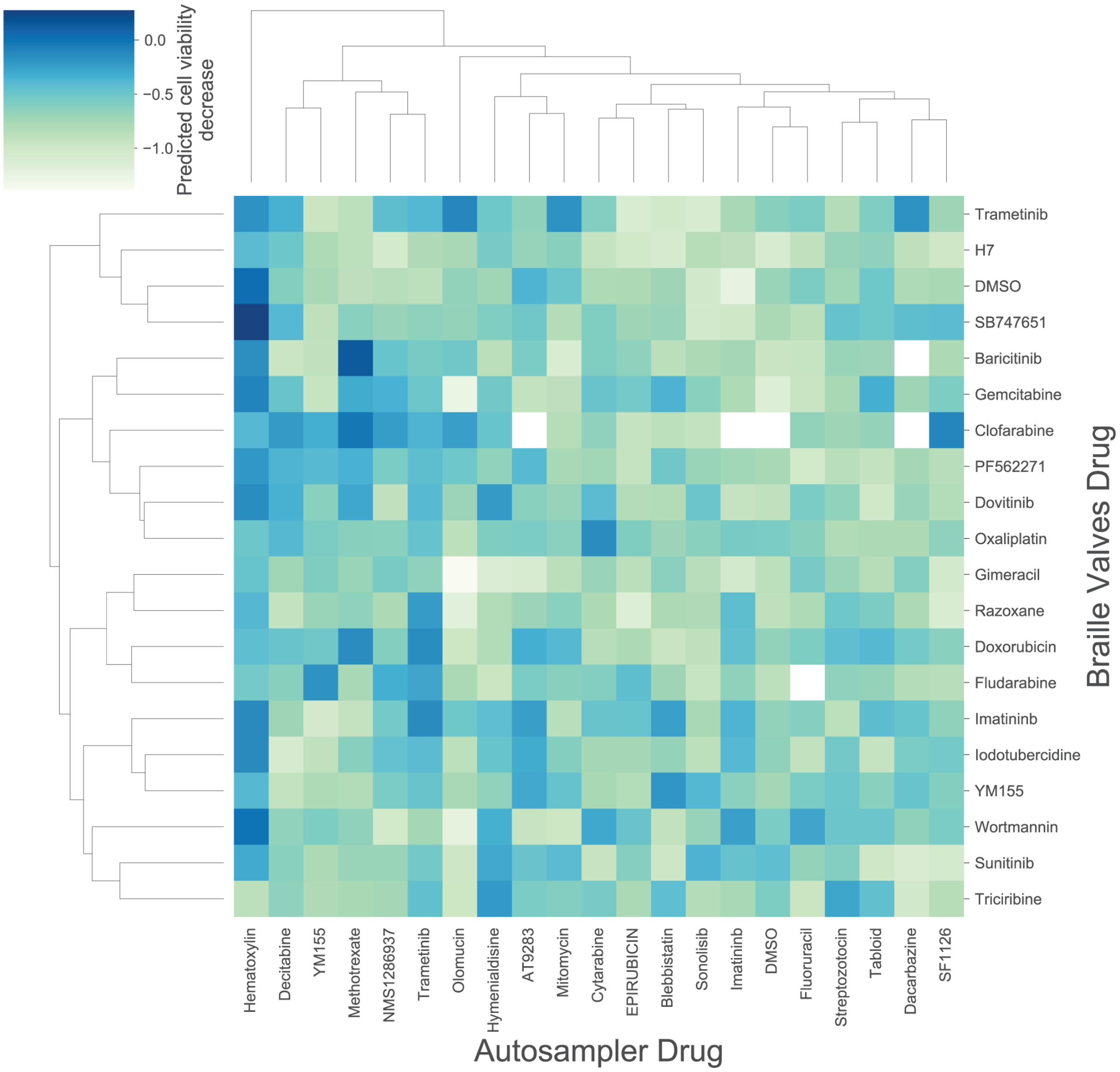

Supplementary Fig. 18: Heatmap of predicted cell viability for drug combinations. Cell viability was predicted from gene expression data using the CEVIChE method. Source data are provided as a Source Data file.

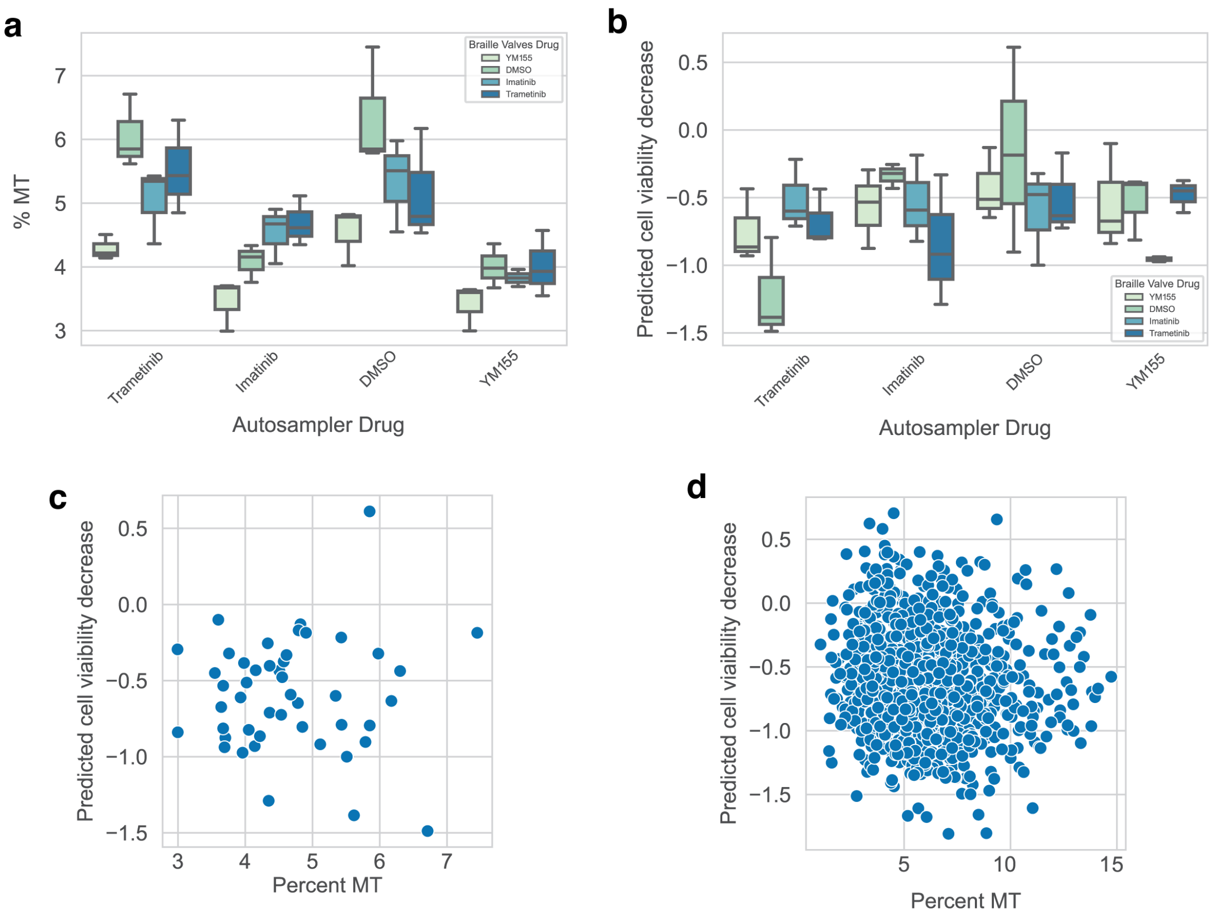

Supplementary Fig. 19: Impact of mitochondrial gene detection on predicted cell viability. (**a**) Percentage of mitochondrial genes detected (%MT) for samples of the 4x4 screen, n = 3 biological independent experiments for all samples, except for YM155_Imatinib and DMSO_Imatinib n = 2. The box plots show the median and first and third quartiles as a box, and the whiskers indicate the most extreme data points within 1.5 lengths of the box. (**b**) Decrease in the predicted cell viability for the same samples as shown in a, n = 3 biological independent experiments for all samples, except for YM155_Imatinib and DMSO_Imatinib n = 2. The box plots show the median and first and third quartiles as a box, and the whiskers indicate the most extreme data points within 1.5 lengths of the box. (**c**) Scatterplot of % MT and predicted cell viability decreases for the 4x4 screen. (**d**) Scatterplot of % MT and the decrease in the predicted cell viability for the large screen. Source data are provided as a Source Data file.

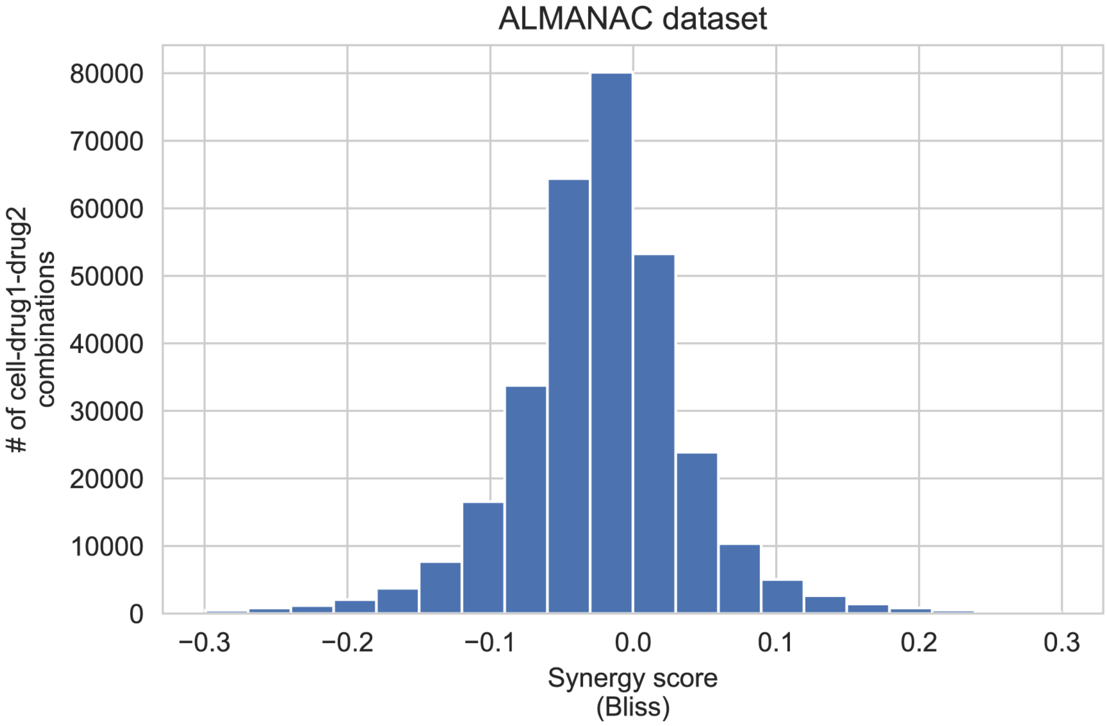

Supplementary Fig. 20: Distribution of Bliss synergy scores from the ALMANAC combinatorial drug screen. Source data are provided as a Source Data file.

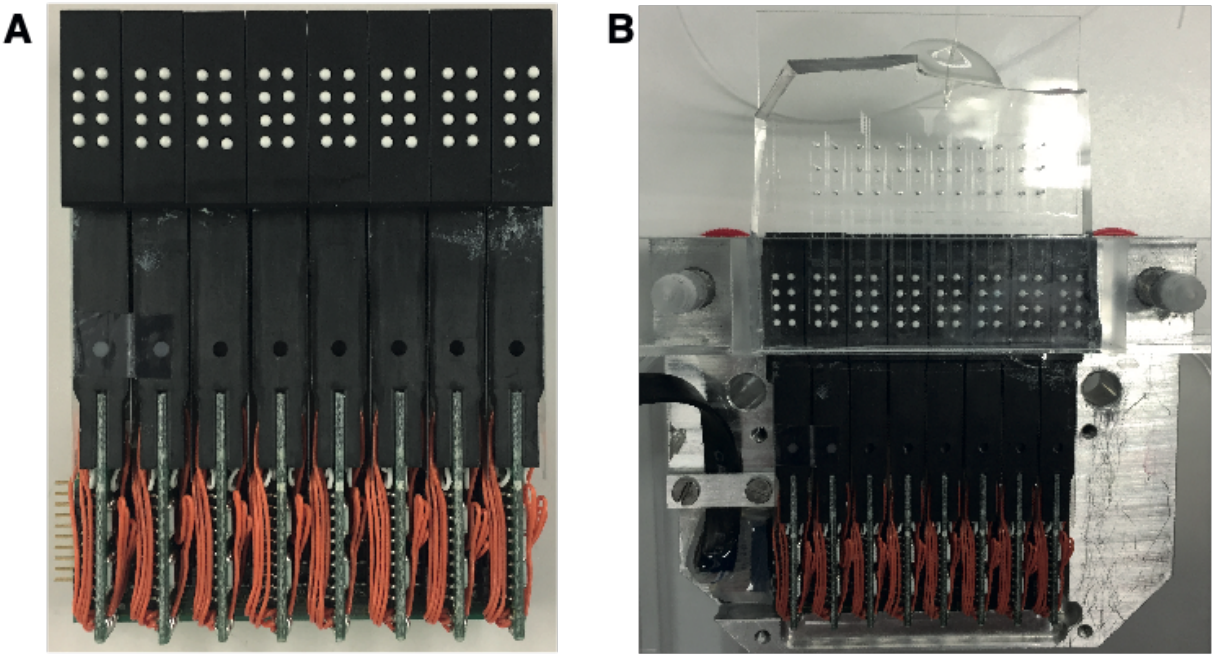

Supplementary Fig. 21: Pictures of the Braille display. (**A**) Braille display alone. (**B**) Braille display mounted on the home-made chip holder. A Braille valve chip was mounted on top so that the Braille pins were aligned with the rectangular valve structures of the chip.

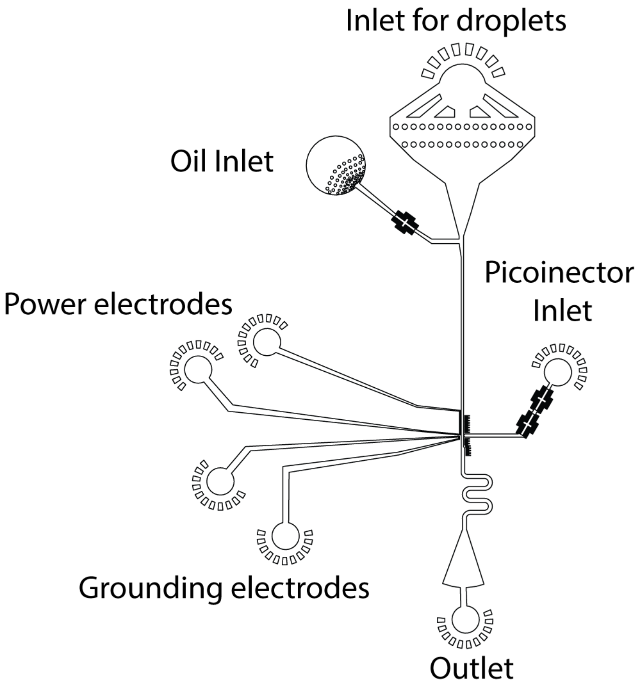

Supplementary Fig. 22: Chip design used to manufacture molds for Pico-injection devices. Channels for the power and grounding electrodes were filled with solder and cables to connect the chip with a function generator or for grounding were inserted into the inlet ports.

Supplementary Table 1

Cross contamination measurements

	Chip 1	Chip 2	Chip 3
Average contamination UV channel	1.44% (n=99)	0.98% (n=80)	2.3% (n=171)
Average contamination green channel	0.92% (n=99)	0.47% (n=80)	0.73% (n=171)

Supplementary Table 2

Correlations between predicted and measured synergy scores

Synergy dataset	Pearson r, r2, p	Spearman r, p
w/ razoxane x trametinib	0.67, 0.44, **0.018**	0.59, **0.041**
w/o razoxane x trametinib	0.65, 0.42, **0.031**	0.48, *0.133*

Supplementary Table 3

Oligonucleotides used for the preparation of sequencing libraries

Sequence Name	Sequence (5' -> 3')
TSO	AAGCAGTGGTATCAACGCAGAGTGAATrGrGrG
SMART-Primer	AAGCAGTGGTATCAACGCAGAGT
Tn5ME (loaded on Tn5)	GTCTCGTGGGCTCGGAGATGTGTATAAGAGACAG
Tn5MErev	[phos]CTGTCTCTTATACACATCT
i7 indexed P7 adapter primers	CAAGCAGAAGACGGCATACGAGATnnnnnnnnGTCTCGTGGGCTCGG
P5-SMART adapter primer	AATGATACGGCGACCACCGAGATCTACACGCCTGTCCGCGGAAGCAGTGGTATCAACGCAGAGTAC
Custom Sequencing Primer	GCCTGTCCGCGGAAGCAGTGGTATCAACGCAG AGTAC

Supplementary Table 4

PCR programs used for the preparation of sequencing libraries

**Whole transcriptome amplification**		
**Step**	**Temperature**	**Time**
Initial denaturation	95 °C	3 min
4 cycles	98 °C<br>65 °C<br>72 °C	20 sec<br>45 sec<br>3 min
9 cycles	98 °C<br>67 °C<br>72 °C	20 sec<br>20 sec<br>3 min
Final extension	72	5 min
Hold	4 °C	
**Tagmentation PCR**		
**Step**	**Temperature**	**Time**
Initial denaturation	95 °C	30 sec
12 cycles	98 °C<br>58 °C<br>72 °C	20 sec<br>15 sec<br>30 sec
Final extension	72	3 min
Hold	10 °C	
