# Supplementary material for: Combi-seq for multiplexed transcriptome-based profiling of drug combinations using deterministic barcoding in single-cell droplets
Source: Nat Commun. 2022 Aug 1;13:4450. doi: 10.1038/s41467-022-32197-0 (PMC9343464; doi:10.1038/s41467-022-32197-0)
Supplement: Supplementary file 2 — Reporting Summary [file 41467_2022_32197_MOESM2_ESM.pdf]

nature portfolio

Corresponding author(s): Christoph Merten

Last updated by author(s): 27.06.2022

Reporting Summary

Nature Portfolio wishes to improve the reproducibility of the work that we publish. This form provides structure for consistency and transparency in reporting. For further information on Nature Portfolio policies, see our Editorial Policies and the Editorial Policy Checklist.

Statistics

For all statistical analyses, confirm that the following items are present in the figure legend, table legend, main text, or Methods section.

n/a	Confirmed	
☐	☒	The exact sample size (*n*) for each experimental group/condition, given as a discrete number and unit of measurement
☐	☒	A statement on whether measurements were taken from distinct samples or whether the same sample was measured repeatedly
☐	☒	The statistical test(s) used AND whether they are one- or two-sided<br>*Only common tests should be described solely by name; describe more complex techniques in the Methods section.*
☐	☒	A description of all covariates tested
☐	☒	A description of any assumptions or corrections, such as tests of normality and adjustment for multiple comparisons
☐	☒	A full description of the statistical parameters including central tendency (e.g. means) or other basic estimates (e.g. regression coefficient) AND variation (e.g. standard deviation) or associated estimates of uncertainty (e.g. confidence intervals)
☐	☒	For null hypothesis testing, the test statistic (e.g. *F*, *t*, *r*) with confidence intervals, effect sizes, degrees of freedom and *P* value noted<br>*Give P values as exact values whenever suitable.*
☒	☐	For Bayesian analysis, information on the choice of priors and Markov chain Monte Carlo settings
☒	☐	For hierarchical and complex designs, identification of the appropriate level for tests and full reporting of outcomes
☒	☐	Estimates of effect sizes (e.g. Cohen's *d*, Pearson's *r*), indicating how they were calculated

Our web collection on statistics for biologists contains articles on many of the points above.

Software and code

Policy information about availability of computer code

Data collection	All fluorescence intensities were measured using the described microfluidics workstation and recorded using in house LabView 2013 software called PlugAcquisition and DropletAcquisition. For visualization, the data was processed in R.<br>RNA-Seq data was collected using NextSeq500 Illumina machine in the paired-end mode. Treatment conditions were demultiplexed using barcodes of read 1.
Data analysis	Data analysis was performed with Python 3. 7, using basic data analysis libraries (pandas, numpy, statsmodels) and Scanpy for RNAseq analysis. Code available under https://github.com/bence-szalai/Combi-Seq-analysis

For manuscripts utilizing custom algorithms or software that are central to the research but not yet described in published literature, software must be made available to editors and reviewers. We strongly encourage code deposition in a community repository (e.g. GitHub). See the Nature Portfolio guidelines for submitting code & software for further information.

Data

Policy information about availability of data

All manuscripts must include a data availability statement. This statement should provide the following information, where applicable:

- Accession codes, unique identifiers, or web links for publicly available datasets
- A description of any restrictions on data availability
- For clinical datasets or third party data, please ensure that the statement adheres to our policy

Dataset from RNA-sequencing experiments are available through GEO over the accession GSE174696:
https://www.ncbi.nlm.nih.gov/geo/query/acc.cgi?acc=GSE174696

Information about chemical compounds used in this study was taken from ChEMBL

Field-specific reporting

Please select the one below that is the best fit for your research. If you are not sure, read the appropriate sections before making your selection.

☒ Life sciences ☐ Behavioural & social sciences ☐ Ecological, evolutionary & environmental sciences

For a reference copy of the document with all sections, see nature.com/documents/nr-reporting-summary-flat.pdf

Life sciences study design

All studies must disclose on these points even when the disclosure is negative.

Sample size	For each drug combination perturbation we performed 3 biological replicates both in the small and the large scale screen.
Data exclusions	Fig. 2d: Short fluorescence peaks generally generated from split plugs were removed, in order to estimate the cross contaminations. Fig. 2e/2f: UV fluorescence signals were thresholded at an intensity of 0.2
Replication	All replicated studies are reported in the manuscript.
Randomization	No randomization was performed. Drugs were assigned to the injection system (Braille valve or autosampler) based on GR35 values. Covariates were controlled out by injecting the same drug from both system and comparing its effect.
Blinding	Blinding was not appropriate for this study design.

Reporting for specific materials, systems and methods

We require information from authors about some types of materials, experimental systems and methods used in many studies. Here, indicate whether each material, system or method listed is relevant to your study. If you are not sure if a list item applies to your research, read the appropriate section before selecting a response.

Materials & experimental systems

n/a	Involved in the study	
☒	☐	Antibodies
☐	☒	Eukaryotic cell lines
☒	☐	Palaeontology and archaeology
☒	☐	Animals and other organisms
☒	☐	Human research participants
☒	☐	Clinical data
☒	☐	Dual use research of concern

Methods

n/a	Involved in the study	
☒	☐	ChIP-seq
☒	☐	Flow cytometry
☒	☐	MRI-based neuroimaging

Eukaryotic cell lines

Policy information about cell lines

Cell line source(s)	K562 cells from ATCC (CCL-243)
Authentication	Authenticated cell lines were bought
Mycoplasma contamination	Cells were tested negative for mycoplasma contamination were kept in aliquots at -80 degrees Celsius. For drug screens, cells not older than 2 months were used.
Commonly misidentified lines (See ICLAC register)	No commonly misidentified cell lines were used in this study
