# Supplementary material for: Combi-seq for multiplexed transcriptome-based profiling of drug combinations using deterministic barcoding in single-cell droplets
Source: Nat Commun. 2022 Aug 1;13:4450. doi: 10.1038/s41467-022-32197-0 (PMC9343464; doi:10.1038/s41467-022-32197-0)
Supplement: Supplementary file 3 — Description of Additional Supplementary Files [file 41467_2022_32197_MOESM3_ESM.pdf]

Title: Supplementary Data 1:
Description: A list of all drugs used in this study

Title: Supplementary Move 1:
Description: Movie showing encapsulation of cells into droplet generated from drug-barcode mixtures

Title: Supplementary Move 2:
Description: Equilibration of the flow after plugs injection

Title: Supplementary Move 3:
Description: Picoinjection of reagents for cell lysis, barcode ligation and reverse transcription
